# Development and evaluation of physiologically based pharmacokinetic drug-disease models for predicting captopril pharmacokinetics in chronic diseases

**DOI:** 10.1038/s41598-021-88154-2

**Published:** 2021-04-21

**Authors:** Muhammad F. Rasool, Shazia Ali, Sundus Khalid, Ramsha Khalid, Abdul Majeed, Imran Imran, Hamid Saeed, Muhammad Usman, Mohsin Ali, Amer S. Alali, Abdullah F. AlAsmari, Nemat Ali, Ali Mohammed Asiri, Fawaz Alasmari, Faleh Alqahtani

**Affiliations:** 1 Department of Pharmacy Practice, Faculty of Pharmacy, Bahauddin Zakariya University, Multan, 60800 Pakistan; 2 Department of Pharmaceutics, Faculty of Pharmacy, Bahauddin Zakariya University, Multan, 60800 Pakistan; 3 Department of Pharmacology, Faculty of Pharmacy, Bahauddin Zakariya University, Multan, 60800 Pakistan; 4 University College of Pharmacy, Allama Iqbal Campus, University of the Punjab, Lahore, 54000 Pakistan; 5 Institute of Pharmaceutical Sciences, University of Veterinary and Animal Sciences, Lahore, Pakistan; 6 Department of Pharmacy Practice, Faculty of Pharmaceutical Sciences, Government College University, Faisalabad, 38000 Pakistan; 7 Department of Pharmaceutics, College of Pharmacy, Prince Sattam Bin Abdulaziz University, Al-Kharj, 11942 Saudi Arabia; 8 Department of Pharmacology and Toxicology, College of Pharmacy, King Saud University, Riyadh, 11451 Saudi Arabia

**Keywords:** Pharmacokinetics, Clinical pharmacology

## Abstract

The advancement in the processing speeds of computing machines has facilitated the development of complex physiologically based pharmacokinetic (PBPK) models. These PBPK models can incorporate disease-specific data and could be used to predict pharmacokinetics (PK) of administered drugs in different chronic conditions. The present study aimed to develop and evaluate PBPK drug-disease models for captopril after incorporating relevant pathophysiological changes occurring in adult chronic kidney disease (CKD) and chronic heart failure (CHF) populations. The population-based PBPK simulator Simcyp was used as a modeling and simulation platform. The visual predictive checks and mean observed/predicted ratios (ratio_(Obs/pred)_) of the PK parameters were used for model evaluation. The developed disease models were successful in predicting captopril PK in all three stages of CKD (mild, moderate, and severe) and CHF, as the observed and predicted PK profiles and the ratio_(obs/pred)_ for the PK parameters were in close agreement. The developed captopril PBPK models can assist in tailoring captopril dosages in patients with different disease severity (CKD and CHF).

## Introduction

The advancement in technology and the processing speeds of computing machines have facilitated the development of complex pharmacokinetic (PK) models, that are making the application of modeling and simulation process easier, faster, and safer^1–3^. The application of physiologically based pharmacokinetics (PBPK) in the pharmaceutical industry has made the drug discovery process swift as it decreases the time required to get information regarding the PK of the novel drugs^4^. The PBPK modeling can not only assist in the development of new drug molecules but by providing opportunities to incorporate drug-related in-vitro data, it can also help in the development of novel drug dosage forms^5^. Furthermore, due to their capacity to incorporate disease-specific data, the PBPK models are also being used for the prediction of PK of different drugs in chronic diseases^6–10^.

Diseases like chronic kidney disease (CKD) and chronic heart failure (CHF) are associated with many pathophysiological changes like alteration in blood flows, decrease in plasma protein concentrations, and decrease in metabolic drug clearance (CL) of eliminating organs (kidney and liver)^11–14^. By incorporating these pathophysiological changes into a PBPK model, we can predict absorption, distribution, metabolism, and elimination (ADME) of administered drugs in CKD and CHF populations. The PBPK model-based predictions can assist in designing clinical trials in these chronic conditions (CKD & CHF) and might assist in determining required drug doses with the disease severity (mild, moderate, and severe). Moreover, the impact of renal and hepatic disease is expected to be highest in drugs that undergo extensive metabolism through the kidney and liver. Therefore, if PBPK models are developed for such extensively metabolized drugs, they can be of great clinical use.

Captopril is an angiotensin-converting enzyme (ACE) inhibitor that is being used for the management of hypertension and congestive heart failure^15^, and due to its effectiveness, it is considered to be the first-line antihypertensive agent^16^. Captopril is well absorbed orally and has a swift onset of action^17–19^. When compared with intravenous administration, after oral administration it has a bioavailability of about 60%^17,20,21^. Its bioavailability reduces to 25–50% with co-administration of food and antacid, but its pharmacological activity remains unchanged^22–24^. The total intravenous clearance (CL_iv_) of captopril in healthy adults is 0.76 ± 0.08 (L/h/kg)^18^ and 86% of the administered dose is excreted in urine^25^. It has been reported that captopril PK are altered in CKD as its CL is reduced significantly in patients with different disease severity^26,27^. This reduction in captopril CL results in an increased area under systemic drug concentration. Moreover, captopril is also effective in treating congestive heart failure. The PK of captopril in CHF patients do not differ significantly from that in healthy adults^17,28^. Therefore, dose adjustment may not be required in CHF, but the administered doses should be adjusted in the presence of CKD.

Keeping in view the PK profile of captopril, if a PBPK model is developed that incorporates the pathophysiological changes occurring in CKD and CHF, it can be used to predict its ADME in patients with different degrees of renal and heart failure. Furthermore, after developing and evaluating the developed captopril PBPK disease model with the available clinical PK disease data, due to its mechanistic nature, it can be extended to other populations (geriatrics, liver cirrhosis, pregnant) with different disease severity and may be used to explore different what if clinical scenarios, such as CKD patients with CHF.

To date, there is only one previous report of a PBPK model that has been used for predicting captopril PK in CKD patients^6^, but the focus of the presented work was to develop a PBPK model for predicting captopril ADME in CKD and CHF populations by using a systematic model-building approach. The use of this model-building approach can help in understanding the underlying differences in captopril PK between healthy and disease populations.

The objectives of this study were, (1) to develop and evaluate PBPK drug-disease models for captopril after incorporating relevant pathophysiological changes occurring in CKD and CHF, (2) to predict and compare captopril PK in adult healthy, CKD, and CHF populations.

## Materials and methods

### Modeling software

The Simcyp simulator version 19 (Certara UK Limited, Simcyp Division, Sheffield, UK) was used for modeling and simulation of captopril ADME in healthy and disease populations^29^.

### Modeling strategy

**Figure 1:**
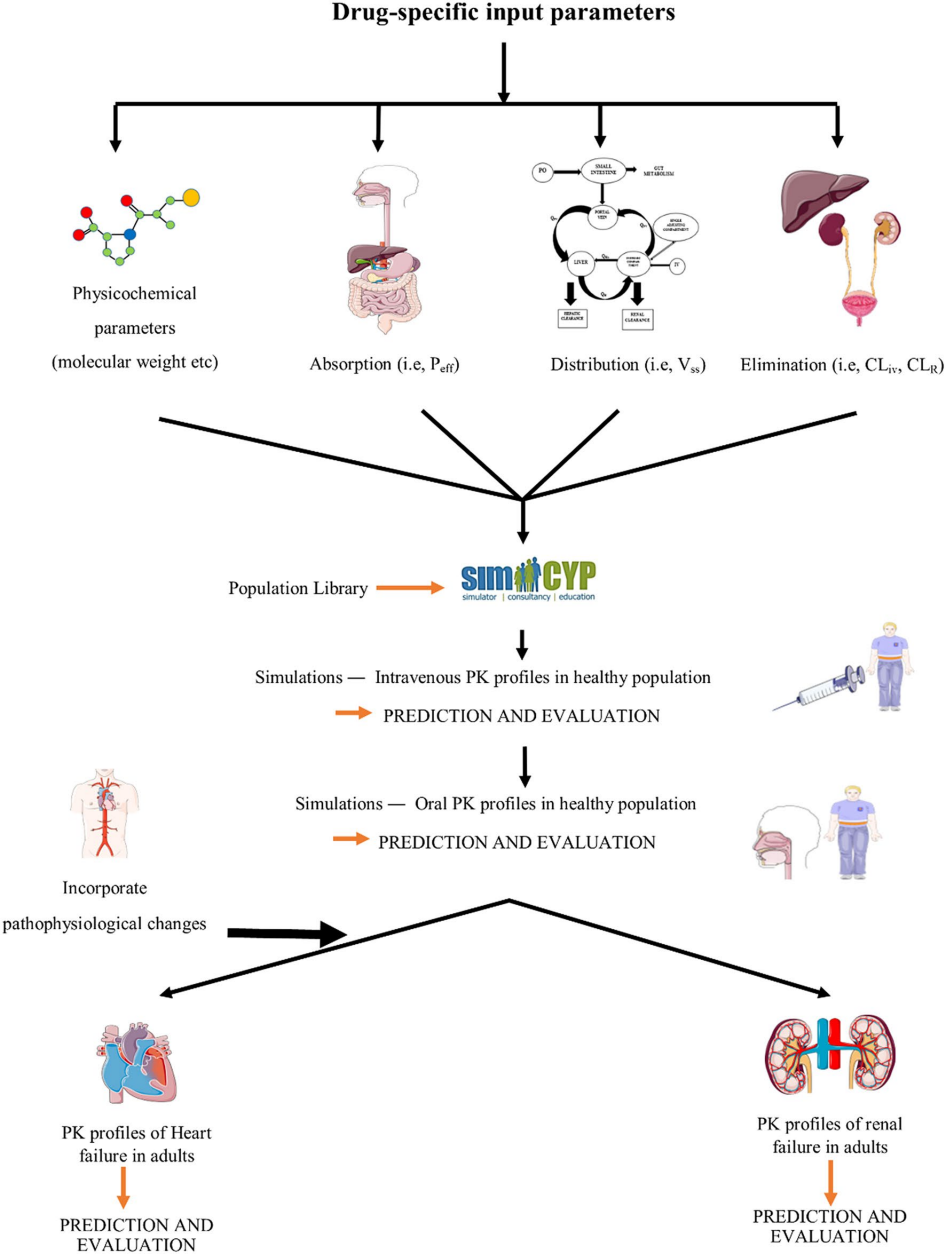
Systematic flow chart diagram for the developed captopril PBPK models in healthy and disease, populations. P_eff_: human jejunum permeability, V_ss_: volume of distribution at steady-state, CL_iv_: intravenous clearance, CL_R_: renal clearance, PK: pharmacokinetics.

The model development process was initiated with an extensive online literature search for identifying and collecting drug-related model input parameters. Based on the presence of clear information on participants, PK data, and the PK profiles of captopril in literature, a total of 9 studies in a healthy population with 15 PK profiles (intravenous = 4 and oral = 11) were selected for model evaluation. Each PK profile consisted of a separate systemic captopril concentration vs. time plot. Two studies were selected in CKD and CHF with 3 and 1 PK profiles, respectively (for details see: Clinical Pharmacokinetic Data). The development and verification of the model was based on the previously used systematic model-building strategy^30,31^. In this adopted strategy, the model development process is started with predictions after intravenous (iv) drug application in healthy adults, so that the complexities associated with modeling of oral drug absorption process can be avoided and parameters that govern the distribution and elimination of the drug can be selected. After successful evaluation of the iv data with the reported PK data, predictions are made after oral drug application and the parameters that control the oral drug absorption process are selected. After a successful evaluation of the developed model in healthy adults, the disease-specific pathophysiological changes are incorporated in the model to constitute the disease model, and then it is used to predict drug PK in disease populations. The workflow for the development and evaluation of the developed PBPK models can be seen in Fig. 1.

### Model structure and parameterization

The compound file created within Simcyp was based on values from the published literature. The final drug-specific input parameters that were used in the developed model can be seen in Table 1. The detailed model parameterization is given below.

**Table 1:** Drug-specific model input parameters used for the development of the captopril PBPK model.

Parameters	Model input values	References
**Physicochemical properties**		
Molecular weight (g/mol)	217.29	^32^
Log*P*_o:w_	0.34	^32^
pK_a_	4.02	^6^
**Absorption**		
**Model: First order**		
k_a_ (1/h)	1.75	Manual optimization*
t_lag_ (h)	0.2	
*f*_a_	0.7	^15^
***Distribution***		
**Model: Minimal PBPK**		
k_in_ (1/h)	0.25	Manual optimization**
k_out_ (1/h)	0.25	
B/P ratio	1	^33^
*f*_u_	0.73	^15^
V_ss_ (L/kg)	0.26	Prediction Method 2 Rodger and Rowland method
**Elimination**		
CL_iv_ (L/h)	49.5	^25^
CL_R_ (L/h)**	22.2	

LogPo:w; octanol–water partition coefficient, ADAM; advanced dissolution, absorption, and metabolism, P_eff_; human jejunum permeability, *f*_u_; fraction of unbound drug in plasma, V_ss_; volume of distribution at steady-state, CL_iv_; intravenous clearance, CL_R_; renal clearance; k_a_; first order absorption rate constant, *f*_a_; fraction of absorbed drug, t_lag_; lag time.

*Manually adjusted after comparing reported and predicted data.

**For details see Supplementary material.

#### Absorption

The first-order absorption model was used to predict the oral drug absorption process. The input values of absorption rate constant and lag time were optimized manually after comparing the reported and predicted clinical PK data. The model input value for the absorbed drug fraction was 0.7^15^. These model input values are consistent with the reported literature as captopril belongs to Biopharmaceutical Classification System (BCS) class I and has a high permeability and solubility^34^.

#### Distribution

The minimal PBPK model was used for predicting captopril distribution. This model is a lumped PBPK model and comprises of four different compartments. This model can be used to predict drug distribution when the volume of distribution is low, and the systemic drug concentration is comparable to the other body tissues. Moreover, due to its simple structure, the simulations in the minimal PBPK model can be performed rapidly. The first-order rate constants, k_in,_ and k_out_ were used to predict drug distribution. The values of k_in_ and k_out_ were selected by manual optimization. The final model input value for k_in_ and k_out_ was 0.25 (1/h). These final input values were derived by the comparison of observed and predicted PK profiles after iv drug administration. The details for this optimization can be seen in Supplementary Methods. The steady-state volume of distribution (${V}_{ss}$) and the tissue-plasma partition coefficients (${K}_{p}$) were predicted by using the method-2 (The Rodgers and Rowland method) implemented within Simcyp^35^. The predicted ${V}_{ss}$ value for captopril was 0.267 L/kg.

#### Elimination

The prediction of drug elimination was based on the total clearance (CL_iv_) and renal clearance (CL_R_)^25^. The model input values for CL_iv_ and CL_R_ can be seen in Table 1.

### Disease-specific pathophysiological changes

#### CKD

The CKD is a quite common condition that is associated with changes in the ADME of administered drugs and it often requires adjustment in administered drug doses^36^. The CKD is not only associated with alteration in the elimination of administered drugs by the renal route, but it also affects the non-renal routes of drug elimination^11^. The changes in glomerular filtration rate (GFR), gastric emptying time, hematocrit and plasma protein binding associated with CKD have been implemented in the Simcyp renal failure populations^11^. There are two renal failure populations within Simcyp which are based on pathophysiological information related to moderate and severe CKD (Sim-RenalGFR_30-60 and Sim-RenalGFR_less_30) that were used to predict captopril ADME in moderate and severe kidney disease. To account for the reduction in non-renal CL in kidney disease populations, as reported in previous PBPK studies scaling factors (SF’s) were used^6,37,38^. The non-renal CL was 20% decreased in the mild kidney disease population, along with the reduction in the GFR. The hepatic SF’s used were within 55–82% and 48–80% in moderate and severe kidney disease^6,38^. In the present study, the hepatic SF’s were adjusted based on a comparison of observed and predicted PK data in moderate and severe kidney disease. The SF’s used for predicting captopril PK in moderate and severe kidney disease were 55% and 35% of the value used in healthy adults. Moreover, to make the predictions in agreement with the observed captopril PK data with the reported data, as reported previously, a SF of 2.51 was used to predict the ${V}_{ss}$ and the ${K}_{p}^{\prime}s$ in the kidney disease populations^6^.

#### CHF

It is known that the blood flow to the organs that are involved in drug elimination is decreased in CHF^39^. These reductions in hepatic blood flow are 76%, 54%, and 46% of normal hepatic flow in mild, moderate, and severe heart failure (HF). The reductions in renal blood flow are 78%, 55%, and 63% of normal renal blood flow in mild, moderate, and severe HF^39^. Moreover, the blood flow to the limbs, skin, fat tissue, muscle, and bone is also reduced in CHF^40^. The blood flow to these parts is reported to be 57%, 44%, and 28% of normal blood flow in mild, moderate, and severe HF^39^. These blood flow reductions have already been incorporated in a PBPK model, that was used to predict drug ADME in CHF patients^12^. The clinical study used for the evaluation of predicted data consisted of a mixed population of patients diagnosed with moderate and severe HF, according to New York Heart Association (NYHA) classification^31^. To account for the decrease in non-renal CL in CHF patients, a hepatic SF of 55% of the value used in healthy adults was used, which was based on a comparison of the observed and the predicted PK profiles of captopril.

### Ethics

All the clinical PK data sets used for model evaluation were extracted from the published literature. The systemic drug concentration versus time profiles were scanned with the GetData Graph Digitizer (version 2.26). Therefore, no ethical approval was required for this work.

### Clinical pharmacokinetic data

A comprehensive online literature review was carried out to search and select the published articles related to the clinical PK of captopril. The final selection was made based on the presence of clear information regarding age, weight, sex, dosing, and a systemic captopril concentration profile. A total of 09 clinical studies in healthy adults with 15 systemic captopril concentration profiles (iv = 4 and oral = 11) in 125 healthy subjects (iv = 26 and oral = 99) were included (Table 2). Two clinical studies were included in the CKD and CHF populations. There were 18 patients and 3 captopril systemic concentration profiles in the CKD study and 20 patients with 01 captopril systemic concentration profiles in the CHF study (Table 3).

**Table 2:** Population data used for development and evaluation of the captopril PBPK model in healthy adults.

Sr. no	Population	No. of subjects	Dose (mg)	Portion of females	Age (year)	Weight (kg)	References
	**iv Bolus**						
1	Healthy	5	10	0	24–34	68.1–88.7	^18^
2	Healthy	7	2.78	0	20–33	–	^25^
		7	5.67	0	20–33	–	
		7	11.4	0	20–33	–	
	**Oral healthy**						
3	Healthy	24	25	0.167	19–25	45–86	^41^
4	Healthy	5	10	0	24–34	68.1–88.7	^18^
5	Healthy	12	25	–	20–24	–	^42^
		12	50	–	20–24	–	^42^
6	Healthy	12 (Male)	100	0	21.88–25.78	67.53–76.85	^43^
		12 (Female)	100	1	19.55–25.61	51.47–60	
7	Healthy	10	100	0	18–35	64.2–85	^19^
8	Healthy	12	100	0	18–33	60–106	^23^
9	Healthy	12	100	0	65–76	63–89	^44^

**Table 3:** Population data used for development and evaluation of the captopril PBPK model in disease populations.

Sr. no	Population	No. of subjects	Dose (mg)	Portion of females	Age (year)	Weight (kg)	References
1	Mild CKD	4	100	0.75	19–60	57–109.5	^27^
	Moderate CKD	6	100	0.33	21–55	54.3–95.6	
	Severe CKD	8	100	0.62	19–62	47.4–90	
2	Chronic heart failure	20	25	–	47–71	–	^45^

*CKD* chronic kidney disease.

### Model evaluation

The simulations were performed by creating virtual populations with a similar demographic and fasting/fed state information, as reported in the reference clinical studies. For every simulation, a virtual population of 100 individuals was created (10 trials of 10 subjects) as it is the most used strategy. The initial model evaluation was done by using visual predictive checks (VPC), in which the mean observed and predicted systemic captopril concentration profiles were overlaid along with the 5th–95th percentiles and minimum, maximum values for the predictions.

The observed and predicted PK-parameters were compared after performing a non-compartmental analysis (NCA) on the observed and predicted systemic captopril concentrations profiles using excel add-in program Pksolver^46^. The PK-parameters, area under the curve (AUC), maximum concentration of drug (C_max_), and the clearance of the drug (CL) were calculated. The observed/predicted ratios (R_obs/Pre_) were also calculated for these PK-parameters with their 95% confidence intervals (Eq. 1). As used in other PBPK model-based studies, a twofold error was used as a reference for model evaluation^47–49^. To assess model accuracy, fold-error, and average fold error (AFE) were also used (Eqs. 2 and 3).

- Ratio (R_obs/pre_)
  1 $$\mathrm{R }=\frac{\mathrm{Observed\; value\;of \;PK\; parameter}}{\mathrm{Predicted \;value \;of\; PK\; parameter}}$$
- Fold-error
  2 $$\mathrm{Fold}-\mathrm{error }=\frac{\mathrm{Observed \;values\; of\; parameter}}{\mathrm{Predicted \;values\; of\; parameter}}$$
- Average fold error (AFE)
  3 $$AFE= {10}^{\frac{\sum \mathrm{log}(fold-error)}{\mathrm{N}}}$$

## Results

### Healthy adults

#### Intravenous application

**Figure 2:**
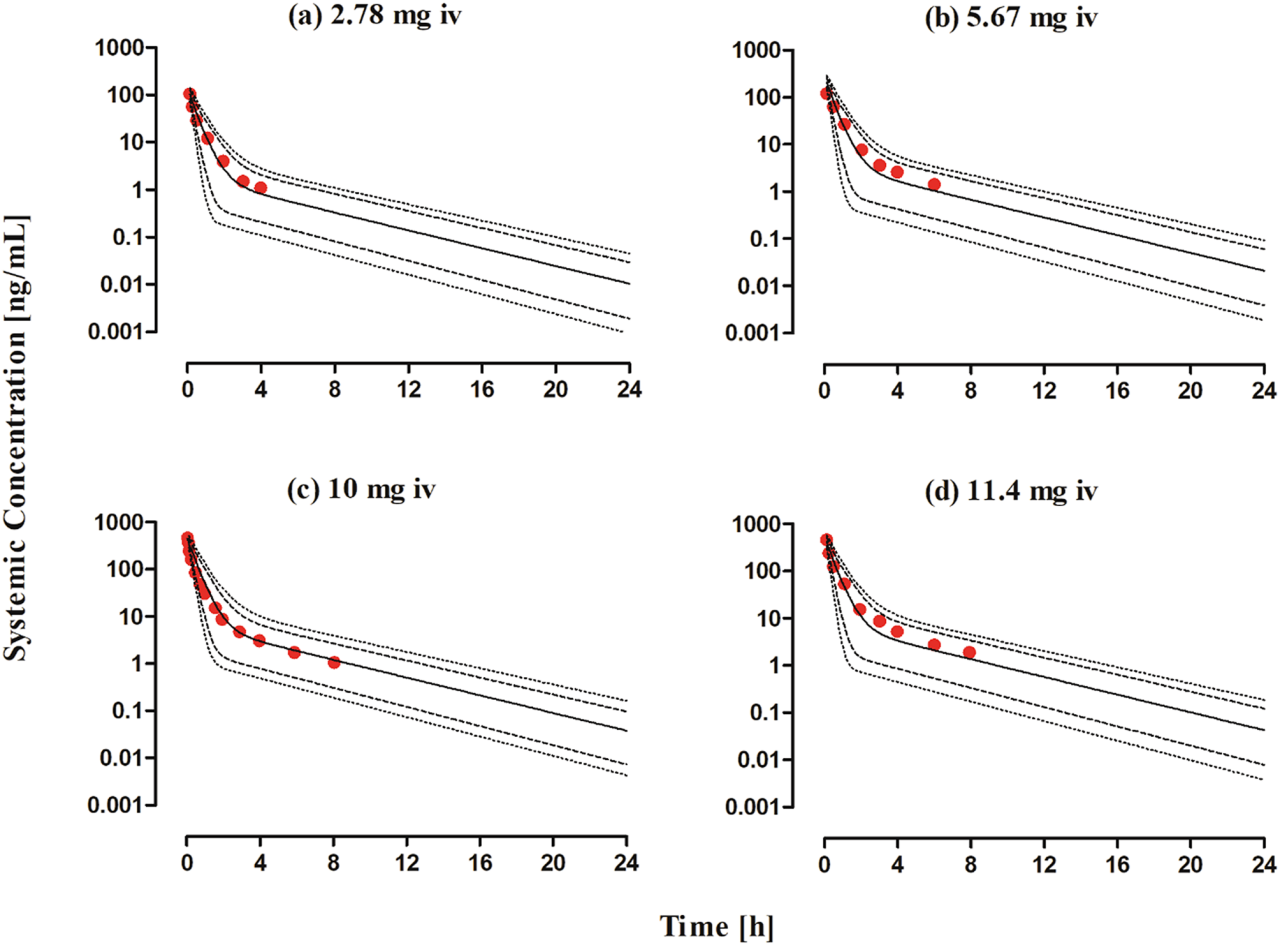
Observed and predicted systemic concentration profiles of captopril in healthy adults after iv bolus administration. (**a**) 2.78 mg, (**b**) 5.67 mg, (**c**) 10 mg^25^ and (**d**) 11.4 mg^18^. The observed data are shown as (red circles). The predicted results are shown as mean (solid line), maximum value, and minimum value (dashed line) and the 5th–95th percentiles (dotted line).

**Figure 3:**
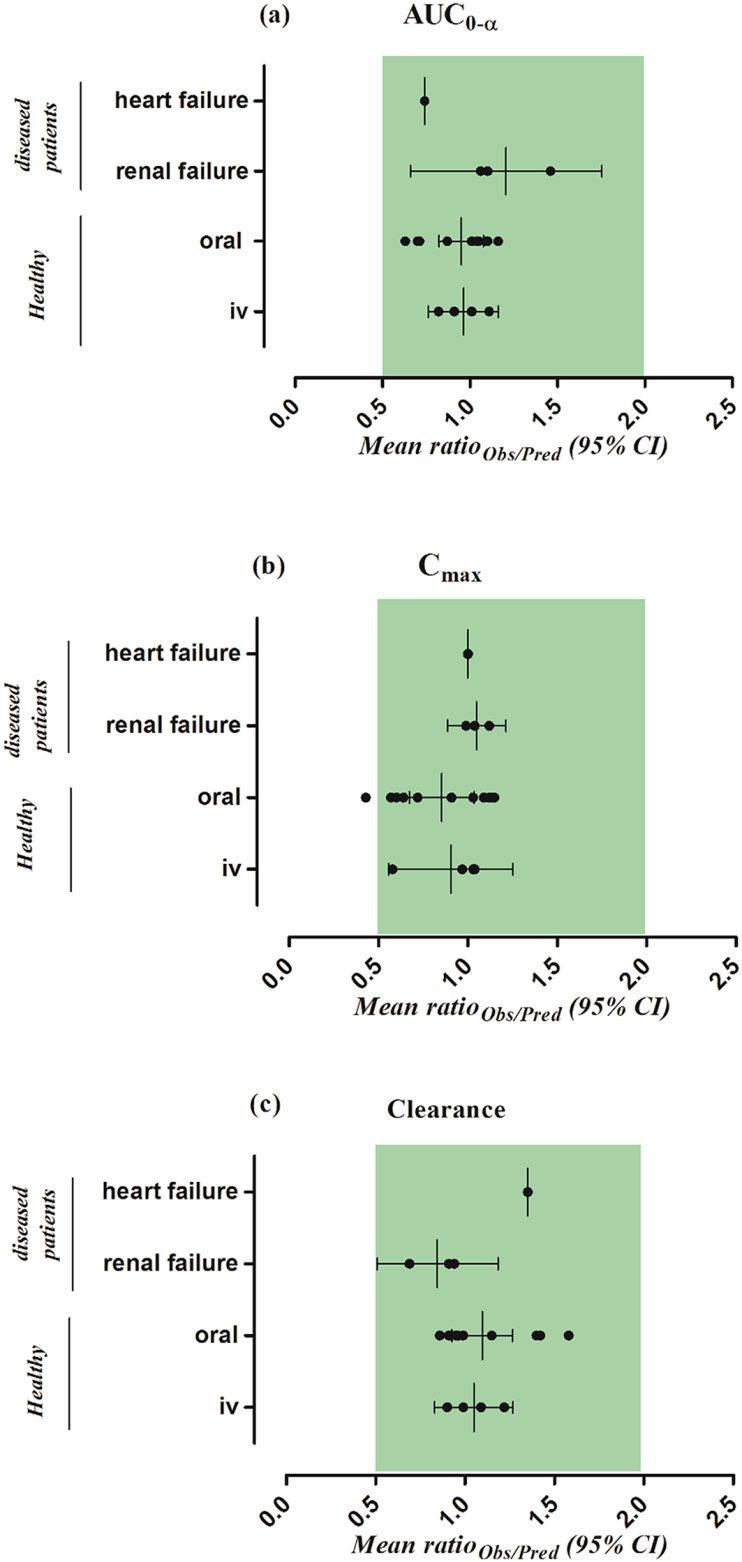
The observed/predicted ratio [ratio_(Obs/Pred)_] for, (**a**) the area under the curve from time 0 to the infinity (AUC_0–∞_), (**b**) the maximum systemic concentration (C_max_ ), and (**c**) the drug clearance (CL) in healthy, renal and heart failure populations. Results are presented as the mean observed/predicted ratio [ratio_(Obs/Pred)_] along with their 95% confidence intervals.

**Figure 4:**
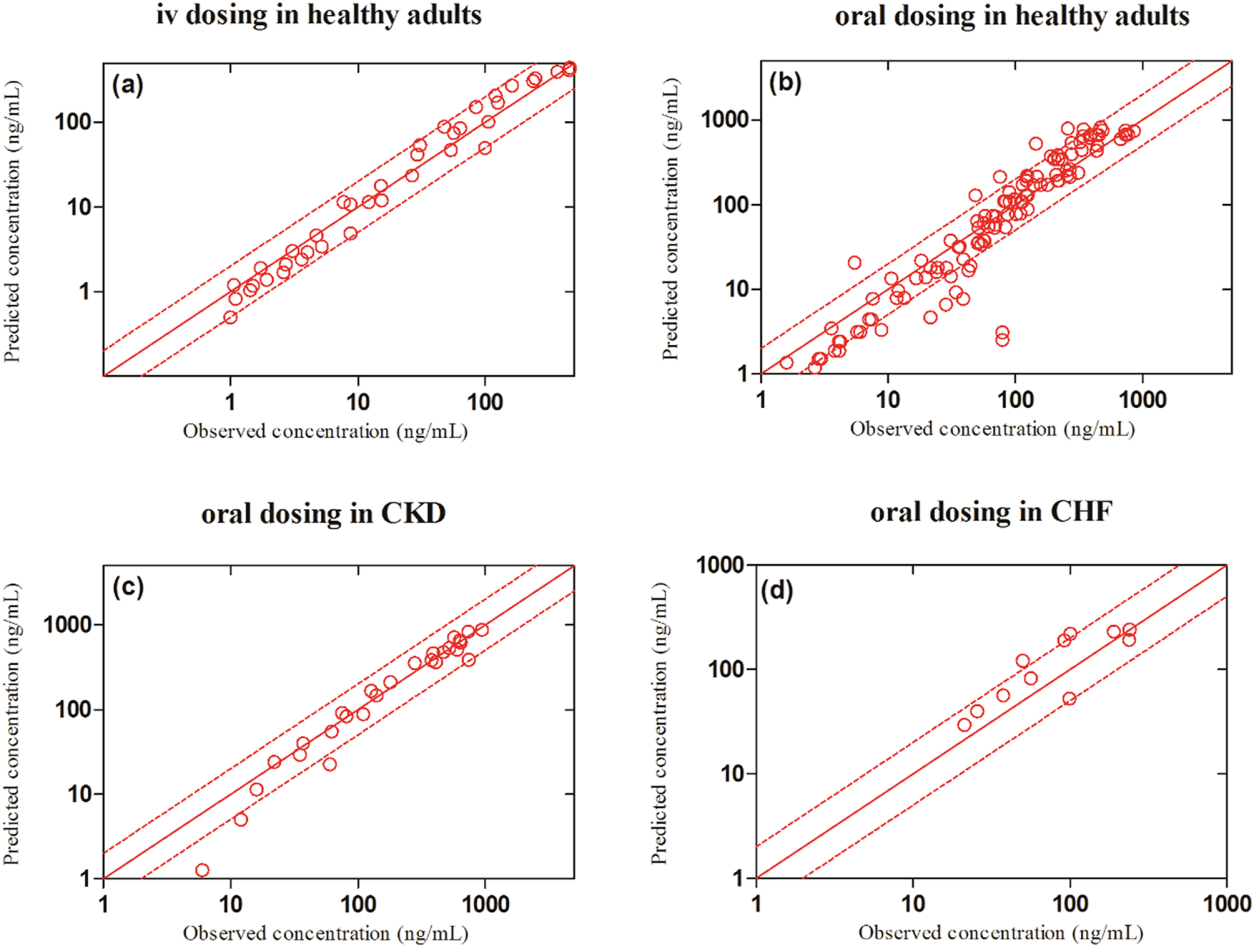
The observed versus predicted captopril systemic concentration plots in healthy and diseased populations. The (red circles) represent the observed vs predicted captopril concentrations. The (red solid line) indicates the line of identity; the (red dashed line) indicates a two-fold error range.

The observed and predicted systemic captopril concentration profiles after iv bolus application within the dose range of 2.78–11.4 mg dose can be seen in Fig. 2. It can be seen from the VPC that the model predictions were in agreement with the observed systemic captopril concentrations at all administered doses. The mean R_obs/pre_ with 95% confidence interval (95% CI) for the AUC, C_max_ and CL were 0.96 (95% CI 0.76–1.16), 0.9 (95% CI 0.55–1.25) and 1.05 (95% CI 0.83–1.26) respectively (Fig. 3 and Table 4). All the predicted systemic captopril concentrations and the PK parameters were within a twofold error range, indicating that the model has adequately predicted captopril PK after iv application and there was no systematic error in the predictions (Table 5 and Fig. 4a).

**Table 4:** The R_obs/pre_ for PK parameters in healthy adults after iv administration of captopril.

PK parameters	Healthy			References
	Observed	Predicted	R_obs/pre_	
**Intravenous administration**				
AUC_0-∞_ (ng/mL.h)				
2.78 mg	67.48	66.81	1.01	^25^
5.67 mg	123.21	134.70	0.91	^25^
10 mg	178.99	218.48	0.82	^18^
11.4 mg	310.82	279.21	1.11	^25^
**CL (L/h)**				
2.78 mg	41.20	41.61	0.99	^25^
5.67 mg	46.01	42.09	1.09	^25^
10 mg	55.86	45.77	1.22	^18^
11.4 mg	36.67	40.83	0.90	^25^
**C**_**max**_ **(ng/mL)**				
2.78 mg	105.61	101.34	1.04	^25^
5.67 mg	120.52	206.91	0.58	^25^
10 mg	461.41	446.00	1.03	^18^
11.4 mg	458.73	471.04	0.97	^25^

**Table 5:** The mean observed/predicted ratio (Ratio_obs/pred_) and AFE values of PK parameters in healthy and disease population after administration of iv and oral captopril.

Parameters	Ratio _obs/pred_	AFE
**IV healthy**		
AUC_0-∞_	0.96	0.96
CL	1.05	1.04
C_max_	0.91	0.88
**Oral healthy**		
AUC_0-∞_	0.95	0.93
CL	1.10	1.07
C_max_	0.85	0.81
**Renal and heart failure (oral)**		
AUC_0-∞_	1.09	1.06
CL	0.97	0.94
C_max_	1.04	1.04

#### Oral application

**Figure 5:**
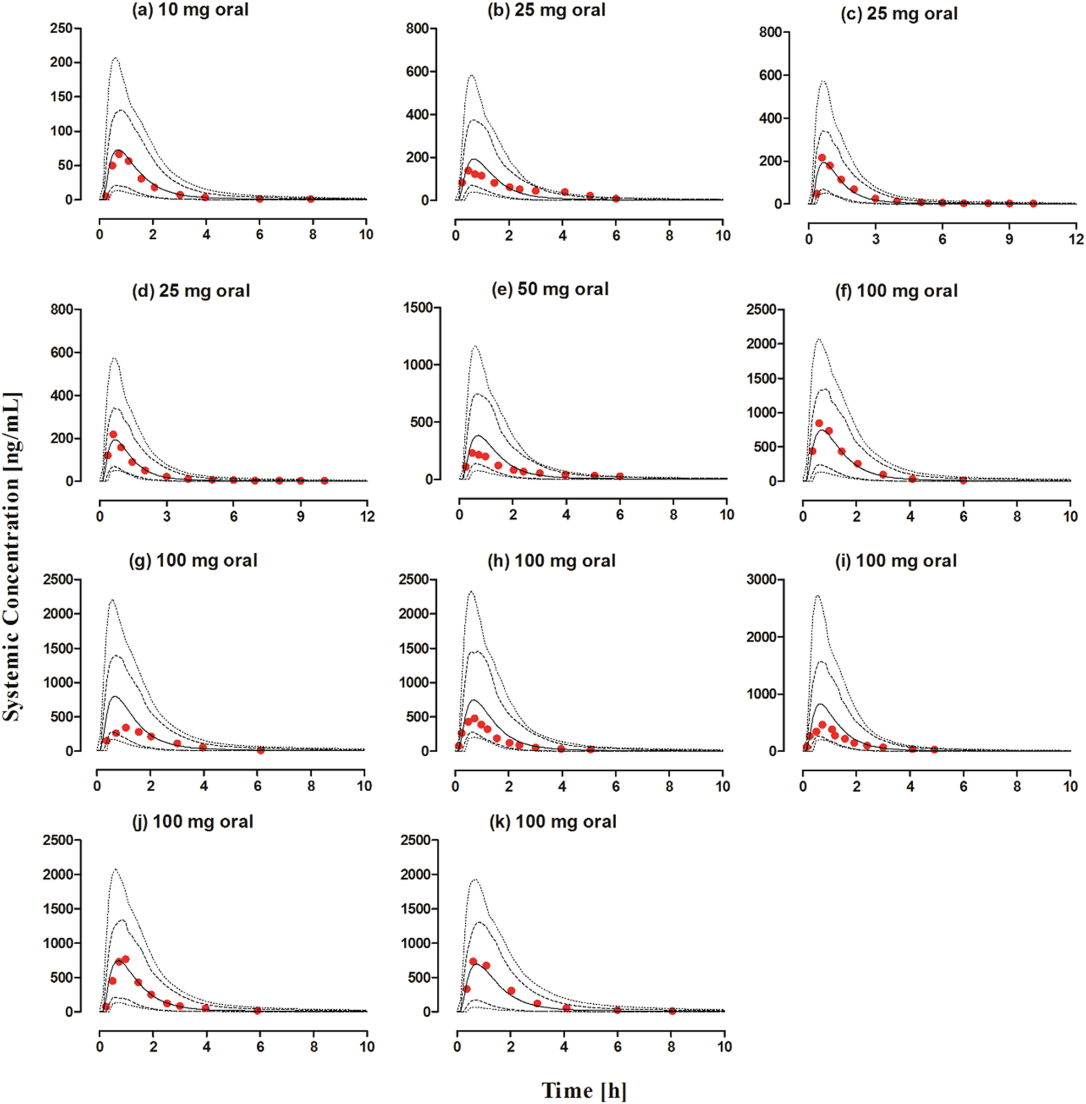
Observed and predicted systemic captopril concentration profiles in healthy individuals after oral administration. (**a**) 10 mg^18^, (**b**) 25 mg^42^, (**c**, **d**) 25 mg^41^, (**e**) 50 mg^42^, (**f**) 100 mg (fasting state), (**g**) 100 mg (fed state)^23^, (**h**) 100 mg (male), (**i**) 100 mg (female)^43^, (**j**) 100 mg^19^, (**k**) 100 mg (geriatrics)^44^. The observed data are shown as (red circles). The predicted results are shown as mean (solid line), maximum value, and minimum value (dashed line) and the 5th–95th percentiles (dotted line).

The systemic captopril concentration profiles for the observed and predicted data after oral application within the dose range of 10–100 mg dose is shown in Fig. 5. The VPC shows that the predictions were in accordance with the observed data at all administered doses. The mean R_obs/pre_ with 95% CI for the AUC, C_max_ and CL/F were 0.95 (95% CI 0.82–1.07), 0.85 (95% CI 0.67–1.03) and 1.09 (95% CI 0.92–1.26) respectively (Fig. 5 and Table 6). There was an increase in the predicted C_max_ of captopril as compared to the fasting state, which was in line with the observed data (Table 6), but no difference in predicted AUC and CL/F was seen. Slight differences were seen in the predicted PK parameters of male and female populations but no such differences were reported in the observed data (Table 6). There was a slight increase in the decrease in captopril CL/F in the geriatric population which was in line with the observed data (Table 6). The majority of the predicted systemic captopril concentrations were within the twofold range (Fig. 4b) and all the PK parameters were within a twofold error range, suggesting that the developed model has accurately predicted captopril PK after oral administration (Tables 5, 6 and Fig. 3).

**Table 6:** The R_obs/pre_ of PK parameters in healthy adults after oral administration of captopril.

PK parameters	Healthy			References
	Observed	Predicted	R_obs/pre_	
**Oral administration**				
AUC_0-∞_ (ng/mL.h)				
10 mg	100.51	115.68	0.87	^18^
25 mg	335.59	304.54	1.10	^42^
25 mg (Test)	348.67	318.55	1.09	^41^
25 mg (Reference)	334.03	317.78	1.05	^41^
50 mg	625.14	603.02	1.04	^42^
100 mg (Fasted)	1339.39	1233.91	1.09	^23^
100 mg (Fed)	782.07	1232.38	0.63	^23^
100 mg (Male)	789.28	1121.55	0.70	^43^
100 mg (Female)	781.54	1094.16	0.71	^43^
100 mg	1198.67	1191.55	1.01	^19^
100 mg (Geriatrics)	1415.87	1223.04	1.16	^44^
**CL/F (L/h)**				
10 mg	99.48	86.45	1.15	^18^
25 mg	74.49	82.09	0.91	^42^
25 mg (Test)	71.70	78.48	0.91	^41^
25 mg (Reference)	74.84	78.67	0.95	^41^
50 mg	79.98	82.92	0.96	^42^
100 mg (Fasted)	74.66	81.04	0.92	^23^
100 mg (Fed)	127.86	81.14	1.58	^23^
100 mg (Male)	126.69	89.16	1.42	^43^
100 mg (Female)	127.95	91.39	1.40	^43^
100 mg	83.42	83.92	0.99	^19^
100 mg (Geriatrics)	70.62	81.76	0.86	^44^
**C**_**max**_ **(ng/mL)**				
10 mg	66.48	72.96	0.91	^18^
25 mg	138.09	192.29	0.72	^42^
25 mg (Test)	215.01	192.29	1.12	^41^
25 mg (Reference)	218.99	192.29	1.14	^41^
50 mg	231.26	384.61	0.60	^42^
100 mg (Fasted)	848.14	739.91	1.15	^23^
100 mg (Fed)	338.75	792.63	0.43	^23^
100 mg (Male)	480.65	750.21	0.64	^43^
100 mg (Female)	466.16	817.07	0.57	^43^
100 mg	768.17	747.28	1.03	^19^
100 mg (Geriatrics)	735.90	676.34	1.09	^44^

### Disease populations

#### Chronic kidney disease

**Figure 6:**
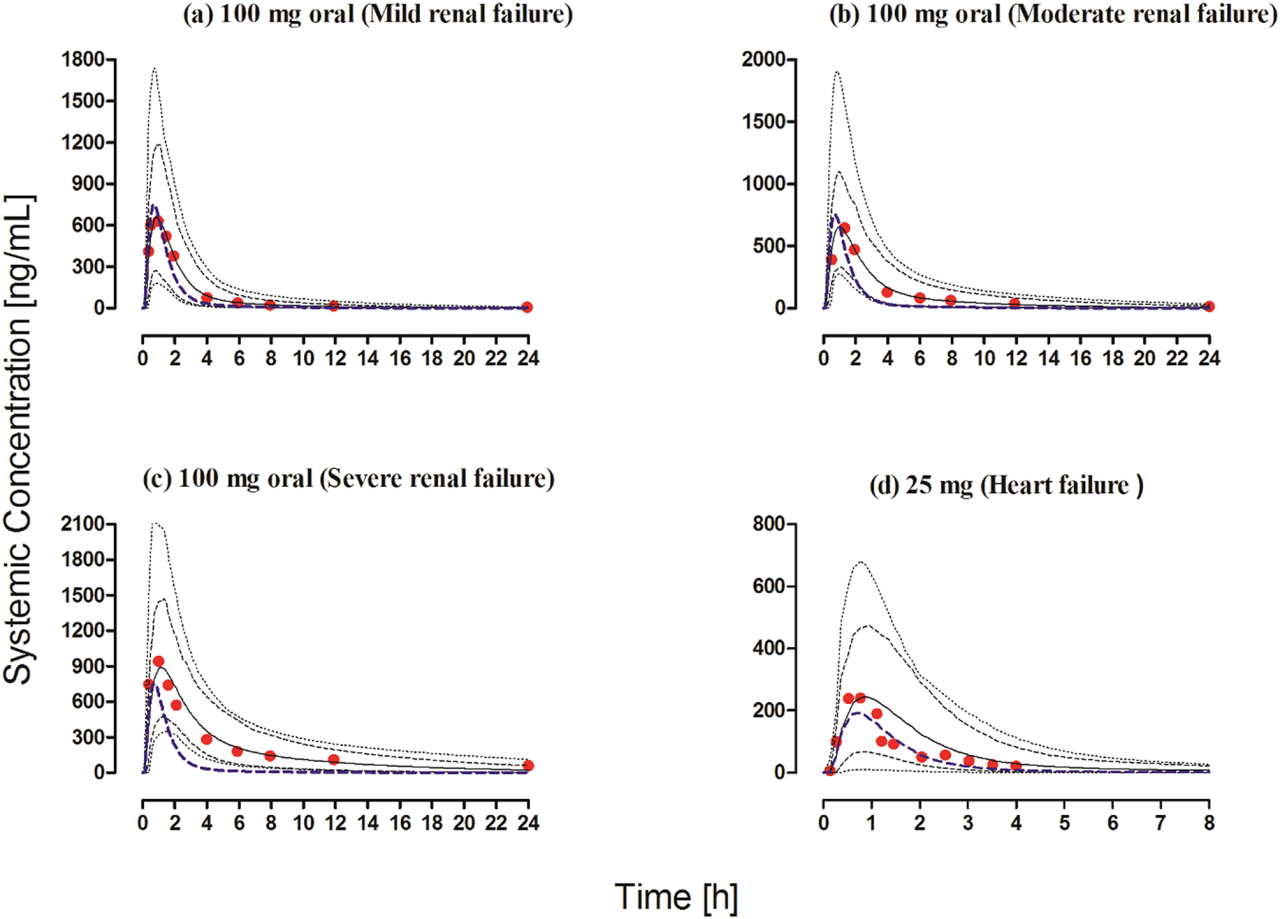
Observed and predicted systemic concentration profiles of captopril in disease populations after oral administration. (**a**–**c**) Mild, moderate, and severe CKD patients after 100 mg oral administration^27^. (**d**) Chronic heart failure patients after 25 mg oral administration^45^. The observed data are shown as (red circles). The predicted results are shown as mean (solid line), maximum value, and minimum value (dashed line) and the 5th–95th percentiles (dotted line). The blue dashed line shows the mean prediction in a healthy population after administering the same oral dose.

The observed clinical data for captopril in CKD populations were within the 5th–95th percentiles of the predicted systemic captopril concentrations in the CKD population after oral administration (Fig. 6a–c). The observed vs. predicted systemic captopril concentration plot showed that most of the predictions were within the twofold range (Fig. 4c). The model has successfully predicted the changes in captopril PK between mild, moderate, and severe CKD populations as all the predicted PK parameters were within a twofold range (Fig. 3, Tables 5, 7).

**Table 7:** R_obs/pre_ of the PK parameters in chronic kidney disease and chronic heart failure populations.

PK parameters	Disease population			References
	Observed	Predicted	R_obs/pre_	
**Oral administration**				
Renal failure				
AUC_0-∞_ (ng/mL.h)				
100 mg (Mild)	1886.24	1715.13	1.10	^27^
100 mg (Moderate)	2469.66	2321.50	1.06	
100 mg (Severe)	6733.48	4612.70	1.46	
CL/F (mg/(ng/L)/h)				
100 mg (Mild)	53.01	58.30	0.91	^27^
100 mg (Moderate)	40.49	43.08	0.94	
100 mg (Severe)	14.85	21.68	0.69	
C_max_ (ng/mL)				
100 mg (Mild)	650	654.72	0.99	^27^
100 mg (Moderate)	644	618.76	1.04	
100 mg (Severe)	981	877.54	1.12	
Heart Failure				
AUC_0-∞_ (ng/mL.h)				
25 mg	375.72	507.41	0.74	^45^
CL/F (L/h)				
25 mg	66.54	49.27	1.35	^45^
C_max_ (ng/mL)				
25 mg	240.21	240.56	1.00	^45^

**Figure 7:**
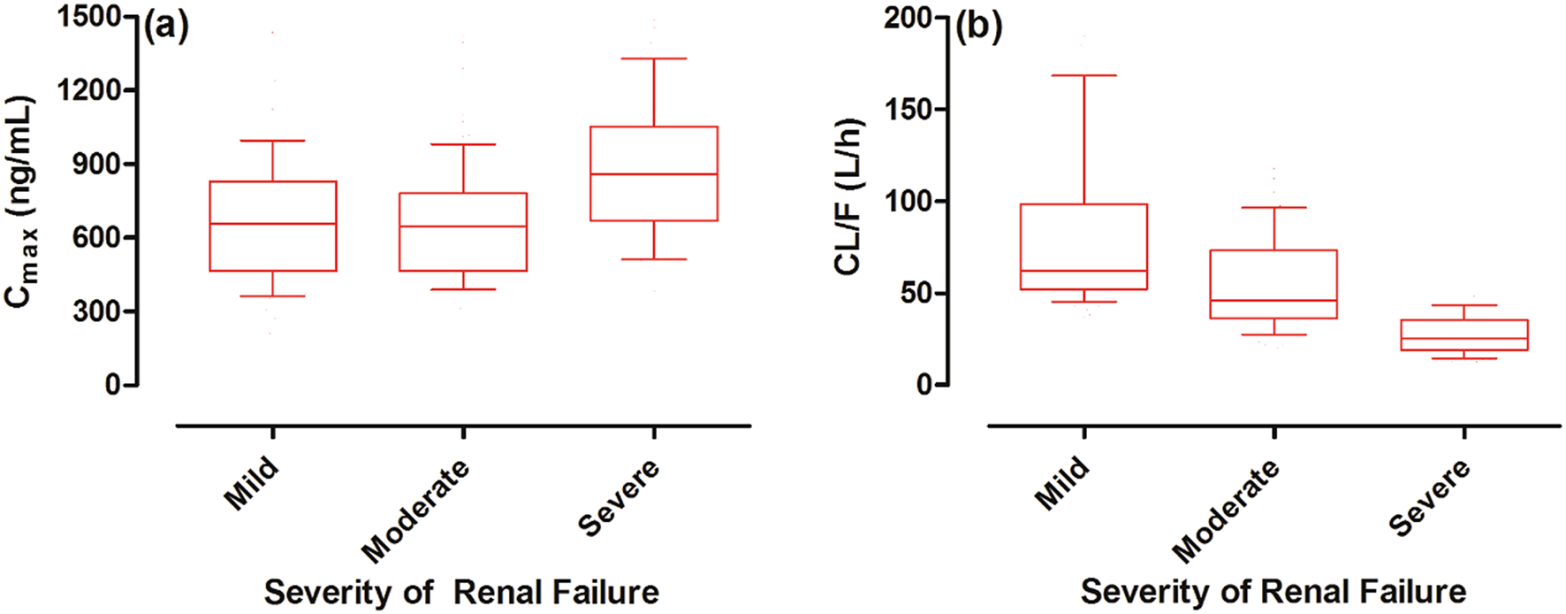
Box plots showing the median, 25th–75th, and 10th–90th percentiles for C_max_ and CL in CKD populations. (**a**) Maximum systemic concentration (C_max_), and (**b**) the oral clearance (CL/F) in mild, moderate, and severe renal failure populations.

The predicted median captopril CL (L/h) decreased with the increase in severity of the disease, as it was 61.87 L/h in mild CKD and was increased to 46.01 L/h in moderate CKD, and was 25.53 (L/h) in severe CKD population (Fig. 7). On the other hand, the increase in predicted median captopril C_max_ (ng/mL) was not so pronounced, as it was 657.8 ng/mL in mild CKD, 645.0 ng/mL in moderate CKD, and 860 ng/mL in severe CKD (Fig. 7).

#### Chronic heart failure

The comparison of observed and predicted systemic captopril concentrations can be seen in the Figs. 4d and 6d. The observed systemic captopril concentrations were within the 5th–95th percentiles of the predicted systemic captopril concentrations and all the predicted PK parameters were within the 2-fold range Tables 5 and 7.

**Figure 8:**
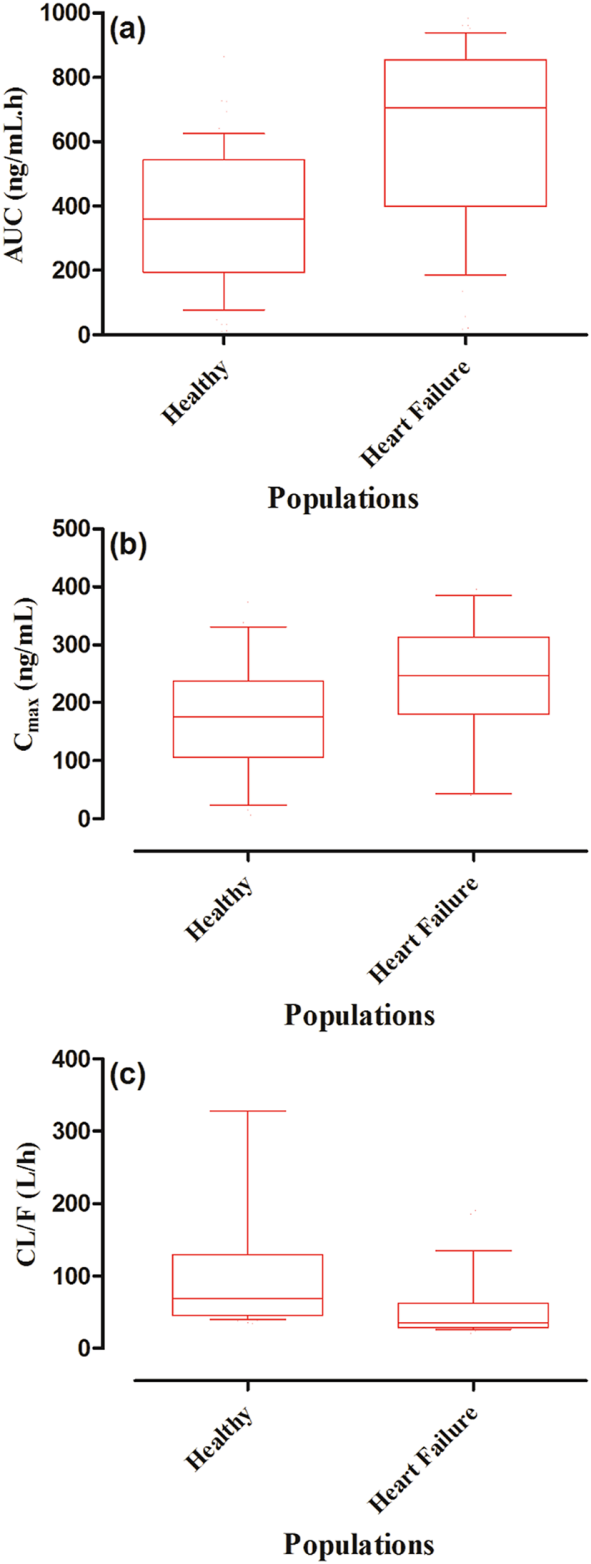
Box plots showing the median, 25th–75th, and 10th–90th percentiles for AUC, C_max,_ and CL in the CHF population. (**a**) Area under the curve from time 0 to the infinity (AUC_0–∞_), (**b**) maximum systemic concentration (C_max_), and (**c**) the oral clearance (CL/F) in healthy and chronic heart failure populations.

There was a noticeable decrease in predicted captopril CL/F in the CHF population (Fig. 8). The predicted median captopril CL/F was 69.43 L/h in healthy adults and was reduced to 35.5 L/h in the CHF population (Fig. 7). Moreover, the predicted median C_max_ (ng/mL) was increased from 176.3 in healthy adults to 246.7 in the CHF population (Fig. 8).

## Discussion

In this study, for the first time, a systematic model building approach was used to develop a PBPK model for predicting captopril ADME in healthy, CKD & CHF populations. The use of this approach has helped in understanding underlying differences in ADME of captopril after iv and oral application in a healthy population. Moreover, after incorporating the disease-associated pathophysiological data in the developed PBPK model, it was used to simulate captopril PK in the disease populations (CKD and CHF). The developed disease models were successful in predicting captopril ADME in all three stages of renal failure (mild, moderate, and severe) and in heart failure.

There has been a previous report of a PBPK model for captopril that was used to predict its PK in healthy and severe CKD patients, and the predictions in moderate CKD were not evaluated against any clinical data^6^. Moreover, this work was focused on predicting the changes in exposure to thiol compounds in CKD patients by constructing PBPK models using the WinNonLin program^6^. In the presented work, PBPK models were developed in adults healthy, CKD and CHF populations by using a population-based ADME simulator, Simcyp. The developed models were successfully evaluated after iv and oral captopril administration in healthy, CKD (mild, moderate, and severe) and CHF populations.

The developed PBPK model has effectively predicted captopril disposition after iv application throughout the dosage range of 2.78–11.4 mg in healthy adults. The AFE for AUC and C_max_ was 0.96 and 0.88, which has provided confidence in the selected model input parameters relating to the distribution and elimination of captopril. After a successful evaluation of the iv data, predictions were performed for oral application in healthy adults. The model predictions after oral captopril administration were comparable to the observed clinical data throughout the dose range of 10–100 mg. The mean R_obs/pred_ (95% CI) for the C_max_ and CL/F were 0.85 (95% CI, 0.67–1.03) and 1.09 (95% CI, 0.92–1.26), depicting that the developed PBPK model has successfully described captopril ADME. Moreover, the predicted captopril bioavailability was 0.5 (range, 0.3–0.9), which was comparable to the reported value of 0.65 (range, 0.49–0.76)^50^.

There was a slight decrease in predicted C_max_ and an increase in predicted AUC of captopril in the geriatric population after administration of 100 mg oral captopril that was consistent with the reported data^23,44^. These changes may be attributed to the age-related decrease in organ sizes that results in a decrease in metabolic organ clearance (hepatic and renal)^51^, along with the decrease in cardiac output (organ blood flows) and plasma protein concentrations, all of these physiological changes have been implemented in “Sim-Geriatric population”. Moreover, there were no notable changes in the predicted captopril CL/F between males and females after the administration of 100 mg oral dose, which is consistent with the reported data^43^. Furthermore, it has been reported that a 35–40% decrease in captopril bioavailability occurs when it is taken with food^23^. The developed model was not able to predict the changes in captopril PK occurring in fasted and fed states. Here it is worth mentioning that the food-related changes in captopril bioavailability have minimal effects on its antihypertensive effects^50^.

A decrease in predicted captopril CL/F was seen in CKD patients with increasing severity of disease. The predicted CL/F was 58.3 L/h in mild CKD patients, and it was reduced to 43.08 L/h in moderate CKD patients and was further reduced to 21.68 L/h in severe CKD patients. These reductions in predicted captopril CL/F were comparable to the values in the reference study^27^, as the mean R_obs/pred_ (range) for CL/F in the CKD populations was 0.84 (0.5–1.18). This reduction in captopril CL/F in CKD populations is associated with the incorporated pathophysiological changes in the Simcyp renal failure populations (Sim-RenalGFR_30-60 and Sim-RenalGFR_less_30). These pathophysiological changes include: decrease in hepatic clearance, an increase in the free fraction of the drug due to a reduction in systemic albumin concentration, delayed gastric emptying, and a decreased in CL_R_ of the drug due to a decrease in GFR^11^.

The incorporation of organ blood flow reductions in the CHF population has resulted in a decrease in predicted CL/F, as it was 74.8 L/h in healthy adults^41^, but was reduced to 66.54 L/h in CHF patients^45^ after administering 25 mg oral captopril. The model predictions in the CHF population were comparable to the reported clinical data as the R_obs/pred_ for CL/F and C_max_ were 1.35 and 1.0, respectively. This decrease in observed and predicted captopril CL/F in the CHF population was associated with the reductions in the organ/tissue blood flows, that were incorporated in the developed CHF model^39,40^. The organ/tissue blood flow reductions used in the developed captopril CHF model have been used previously in describing ADME of different drugs in this disease^12,52^. It is pertinent to mention that the changes in captopril PK in the CHF population do not require dosage modifications^17,28^, but it is expected that dose adjustments may be necessary when the CHF patients are having a comorbidity i.e., CKD or liver cirrhosis.

## Limitations

All the systemic captopril concentration vs. time data used for model evaluation was extracted carefully by scanning publication plots, although the extracted PK parameters were comparable to the reported PK parameters, but minor variations cannot be completely neglected.

To decrease the predicted non-renal captopril CL in CKD and CHF populations, hepatic SF was used. The values of these SF were derived after comparing reported and predicted clinical PK data.

The developed disease models may not reflect the complexity associated with the pathophysiologies of CKD and CHF, as a semi-mechanistic approach was used in the developed models by dividing the total CL into renal and non-renal parts.

The clinical study used for the evaluation of the developed PBPK model in the CHF population consisted of a mixed population of patients with moderate and severe CHF and therefore, it was not possible to evaluate the CHF model in moderate and severe CHF patients separately.

## Conclusion

The developed PBPK models have successfully described captopril PK in healthy and disease (CKD and CHF) populations. The mechanistic nature of the developed captopril PBPK model in CKD can assist in tailoring captopril dosages in patients with impaired kidney functions according to the U.S. Food and Drug Administration (FDA) and European Medicines Agency (EMA) guidelines^53,54^. Similarly, by incorporating the reductions in organ blood flows, the captopril CHF model can be used to predict captopril exposure in patients with different disease severity.

## Supplementary Information

Supplementary Information.
