# Supplementary material for: Development and evaluation of physiologically based pharmacokinetic drug-disease models for predicting captopril pharmacokinetics in chronic diseases
Source: Sci Rep. 2021 Apr 21;11:8589. doi: 10.1038/s41598-021-88154-2 (PMC8060346; doi:10.1038/s41598-021-88154-2)
Supplement: Supplementary file 1 — Supplementary Information. [file 41598_2021_88154_MOESM1_ESM.pdf]

Supplementary Material

Development and evaluation of physiologically based pharmacokinetic drug-disease models for predicting captopril pharmacokinetics in chronic diseases

Muhammad F. Rasool[1]*, Shazia Ali[2], Sundus Khalid[1], Ramsha Khalid[1], Abdul Majeed[1], Imran Imran[3], Hamid Saeed[4], Muhammad Usman[5], Mohsin Ali[6], Amer S Alali[7], Abdullah F AlAsmari[7], Nemat Ali[8], Ali Mohammed Asiri[8], and Faleh Alqahtani[8]*

[1]Department of Pharmacy Practice, Faculty of Pharmacy, Bahauddin Zakariya University, 60800, Multan, Pakistan

[2]Department of Pharmaceutics, Faculty of Pharmacy, Bahauddin Zakariya University, 60800, Multan, Pakistan

[3]Department of Pharmacology, Faculty of Pharmacy, Bahauddin Zakariya University, 60800, Multan, Pakistan

[4]University College of Pharmacy, Allama Iqbal Campus, University of the Punjab, 54000, Lahore, Pakistan

[5]Institute of Pharmaceutical Sciences, University of Veterinary and Animal Sciences, Lahore
[6]Department of Pharmacy Practice, Faculty of Pharmaceutical Sciences Government College University, 38000, Faisalabad

[7]Department of Pharmaceutics, College of Pharmacy, Prince Sattam bin Abdulaziz University, Al-Kharj 11942, Saudi Arabia

[8]Department of Pharmacology and Toxicology, College of Pharmacy, King Saud University, Riyadh 11451, Saudi Arabia

*** Correspondence: fawadrasool@bzu.edu.pk, Afaleh@ksu.edu.sa**

Supplementary Methods

The minimal physiological based pharmacokinetic drug distribution model (MPDDM)

The MPDDM is one of the models that have been implemented in the program Simcyp. It is a lumped PBPK model that has four compartments and is capable of predicting systemic, portal vein and liver concentrations of the administered drug. This model has a single adjusting compartment (SAC), which is a non-physiological compartment that allows changes in the systemic drug concentration. The MPDDM is usually used for predicting drug distribution of drugs with low volume of distribution (V_d). the structure of the MPDDM can be seen in Supplementary Figure S1.

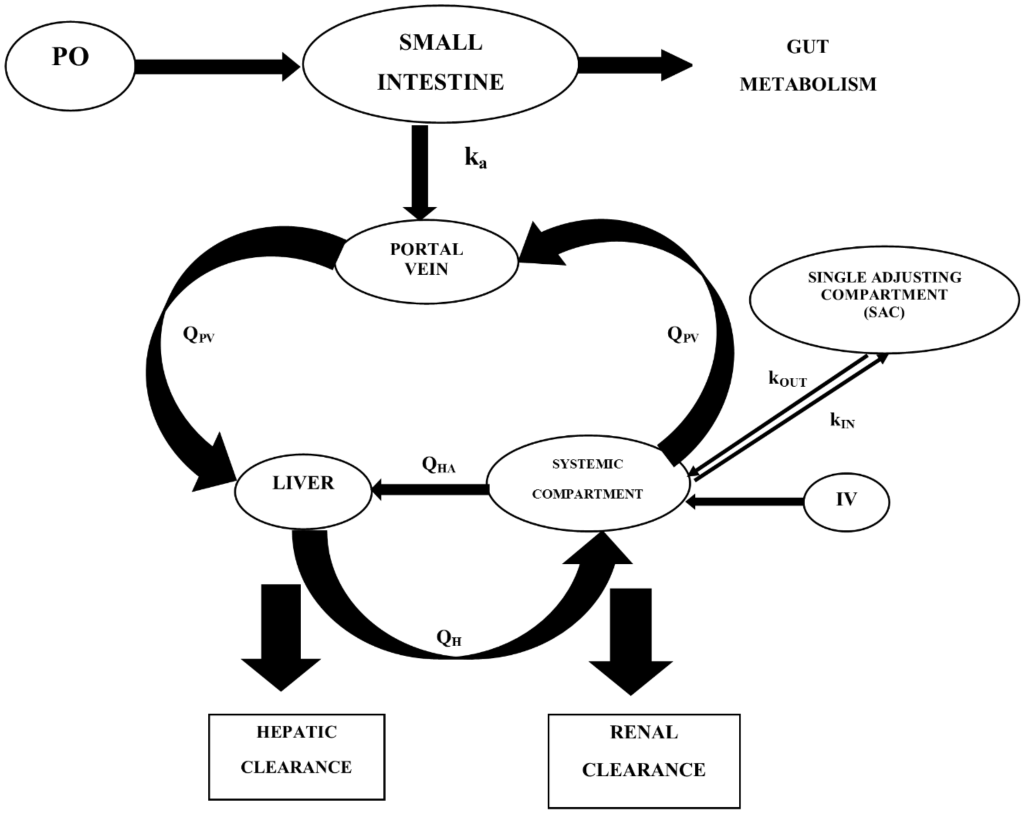

Supplementary Figure S1: Minimal PBPK drug distribution model (MPDDM)

k_{in} and k_{out} are first order rate constants, IV: intravenous, k_a is the absorption rate constant and Q is the blood flow

In MPDDM the apparent volume of the systemic compartment (V_{SYS}) is determined by using the following equation,

$$V_{SYS} = V_{ss} - V_H - V_{SAC}$$

Where V_{SS} is volume of the volume of distribution at steady state, V_H is the apparent hepatic volume and V_{SAC} is the apparent volume related to the SAC.

The users can use input values for k_{in} (or CL_{in}), k_{out} (or CL_{out}) or Q and V_{SAC} for predicting drug distribution in the MPDDM.

The model input values for the first order rate constants, k_{in} and k_{out} were optimized manually by comparing observed and predicted systemic captopril concentration profiles after intravenous (iv) application (Supplementary Table S1). The model input values for k_{in} and k_{out} were changed manually and their initial values were derived after comparing observed and predicted systemic concentrations profiles using visual predictive checks (Supplementary Figure S2). The final selection of model input values for k_{in} and k_{out} (0.25 and 0.25) were based on the comparison of observed and predicted elimination rate constants (k_e), area under the curve (AUC) and maximum systemic concentration (C_{max}) after intravenous captopril administration after iv captopril administration.

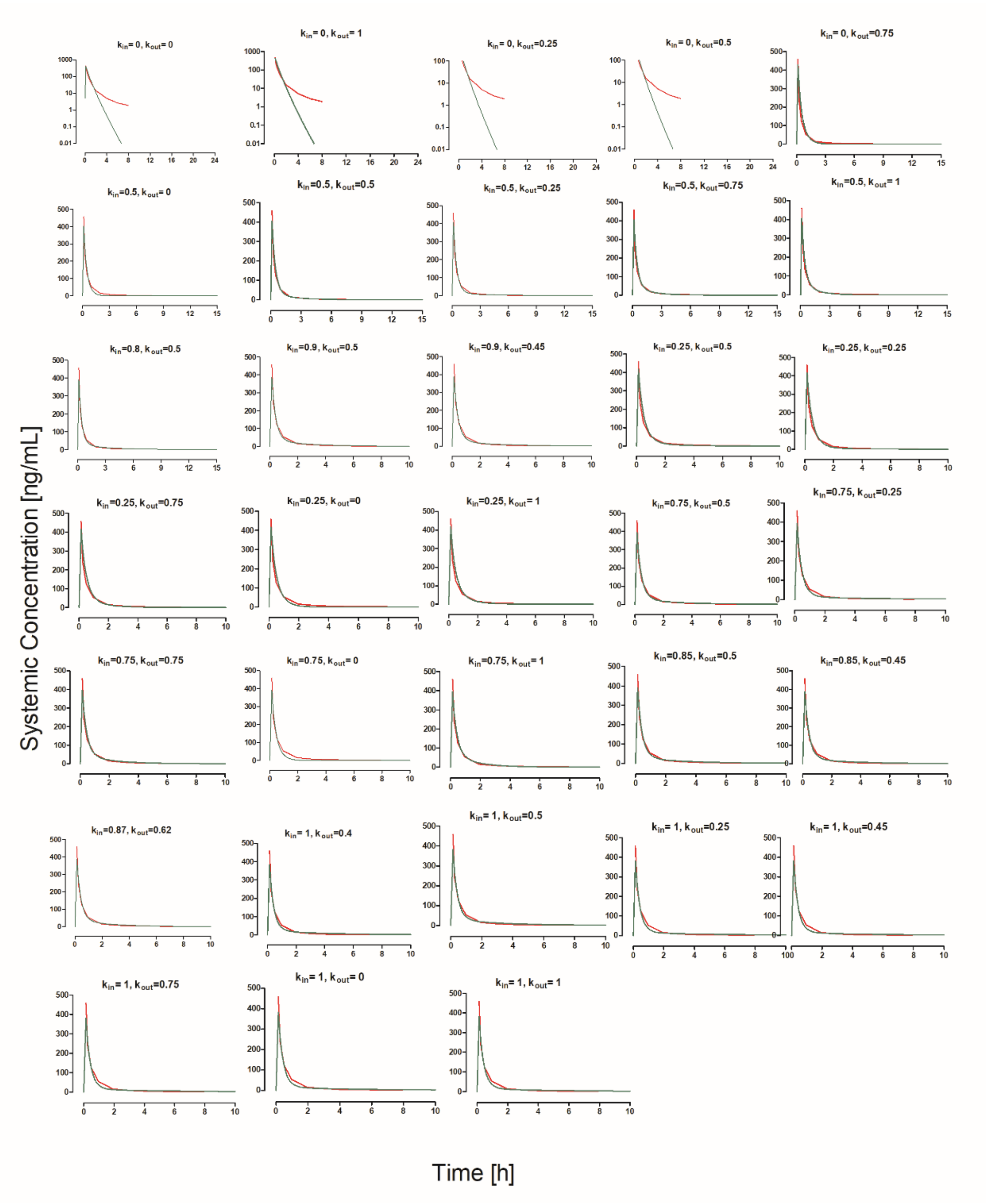

Supplementary Figure S2. Comparison of observed and predicted captopril systemic concentration profiles after intravenous application by changing first order rate constants (k_{in} and k_{out})

Red line shows the observed systemic concentration data after administration of iv captopril [1] *and the green line shows the predicted systemic captopril concentration.*

Supplementary Table S1. Effect of changing first order rate constants on predicted elimination rate constant (k_e), area under the curve (AUC) and maximum systemic concentration C_{max} after intravenous captopril administration.

k_{in}	k_{out}	k_e		AUC_{0-inf}		C_{max}	
		**Observed**	**Predicted**	**Observed**	**Predicted**	**Observed**	**Predicted**
0	0	0.2519	1.3809	279.21	281.5639	458.729	425.5773
0	1		1.4094		279.7714		428.6469
0	0.5		1.3923		280.1926		428.6469
0	0.25		1.3923		280.1926		428.6469
0	0.75		1.3923		280.1926		428.6469
0.5	0		1.8805		236.2172		404.7984
0.5	0.5		0.3726		287.5342		405.6259
0.5	0.25		0.1946		287.2145		405.1810
0.5	0.75		0.5343		287.8020		406.0247
0.5	1		0.6758		287.9879		406.3262
0.8	0.5		0.3286		285.0579		391.9403
0.9	0.5		0.3167		284.2586		387.4984
0.9	0.45		0.2883		287.87		387.38
0.25	0.5		0.4327		288.7976		417.2341
0.25	0.25		0.2286		288.4666		417.0415
0.25	0.75		0.6225		288.8544		417.4398
0.25	0		1.6432		260.2917		416.8124
0.25	1		0.7866		288.8304		417.6314
0.75	0.5		0.3363		287.2020		394.1901
0.75	0.25		0.3373		287.2135		394.1901
0.75	0.75		0.4805		287.3411		394.7606
0.75	0		2.1338		217.9203		393.0449
0.75	1		0.6047		287.3802		395.3318
0.85	0.5		0.3235		286.8941		389.7398
0.85	0.45		0.2937		286.8685		389.5950
0.87	0.62		0.3892		286.8569		389.1598
1	0.4		0.2500		286.2628		382.6409
1	0.5		0.3068		286.3016		382.9126
1	0.25		0.1612		286.1123		382.1795
1	0.45		0.2786		286.3097		382.8418
1	0.75		0.4380		286.4415		383.7049
1	0		2.7669		201.6758		381.1746
1	1		0.5539		286.4486		384.4182

Reference

1 Creasey, W., Morrison, R., Singhvi, S. & Willard, D. Pharmacokinetics of intravenous captopril in healthy men. *European journal of clinical pharmacology* **35**, 367-370 (1988).
